# Rab27a Targeting to Melanosomes Requires Nucleotide Exchange but Not Effector Binding

**DOI:** 10.1111/j.1600-0854.2011.01216.x

**Published:** 2011-06-13

**Authors:** Abul K Tarafder, Christina Wasmeier, Ana C Figueiredo, Antonia E G Booth, Asumi Orihara, Jose S Ramalho, Alistair N Hume, Miguel C Seabra

**Affiliations:** 1 Molecular Medicine, National Heart and Lung Institute (NHLI), Imperial College LondonLondon SW7 2AZ, UK; 3 CEDOC, Faculdade de Ciências Médicas, FCM, Universidade Nova de Lisboa1169-056 Lisboa, Portugal; 4 Instituto Gulbenkian de CienciaOeiras, Portugal

**Keywords:** effectors, guanine nucleotide exchange factor, melanosome, Rab, targeting

## Abstract

Rab GTPases are important determinants of organelle identity and regulators of vesicular transport pathways. Consequently, each Rab occupies a highly specific subcellular localization. However, the precise mechanisms governing Rab targeting remain unclear. Guanine nucleotide exchange factors (GEFs), putative membrane-resident targeting factors and effector binding have all been implicated as critical regulators of Rab targeting. Here, we address these issues using Rab27a targeting to melanosomes as a model system. Rab27a regulates motility of lysosome-related organelles and secretory granules. Its effectors have been characterized extensively, and we have identified Rab3GEP as the non-redundant Rab27a GEF in melanocytes (Figueiredo AC et al. Rab3GEP is the non-redundant guanine nucleotide exchange factor for Rab27a in melanocytes. J Biol Chem 2008;283:23209–23216). Using Rab27a mutants that show impaired binding to representatives of all four Rab27a effector subgroups, we present evidence that effector binding is not essential for targeting of Rab27a to melanosomes. In contrast, we observed that knockdown of Rab3GEP resulted in mis-targeting of Rab27a, suggesting that Rab3GEP activity is required for correct targeting of Rab27a. However, the identification of Rab27a mutants that undergo efficient GDP/GTP exchange in the presence of Rab3GEP *in vitro* but are mis-targeted in a cellular context indicates that nucleotide loading is not the sole determinant of subcellular targeting of Rab27a. Our data support a model in which exchange activity, but not effector binding, represents one essential factor that contributes to membrane targeting of Rab proteins.

Rab proteins, members of the Ras superfamily of small GTPases, act as key regulators of vesicular transport (1). Individual Rabs are exquisitely localized to specific cellular compartments where they are primary determinants of membrane identity and exert multiple local activities (2,3). Rab proteins are post-translationally modified by geranylgeranylation, which is required for reversible membrane association and proper function (4). Furthermore, regulation of Rab function is determined by their ability to cycle between a GDP-bound inactive form and a GTP-bound active form (5). Prenylated Rabs are activated by specific guanine nucleotide exchange factors (GEF) that stimulate the release of GDP and the binding of GTP. Activated GTP-bound Rabs then recruit diverse effector proteins which mediate critical functions in the organization of membrane traffic. GTPase activating proteins (GAPs) return Rabs to their inactive state by accelerating the intrinsic rate of Rab-GTP hydrolysis. Once back in the GDP-bound form, Rabs may be extracted from membranes by Rab guanine dissociation inhibitor (RabGDI) (6).

Understanding the molecular mechanisms determining the specificity of Rab targeting is therefore critical to gain an insight into how the functional identity of organelles is achieved. Several activities have been implicated in this process. The first of these is a putative GDI displacement factor (GDF), which promotes membrane recruitment by destabilizing the interaction between RabGDI and prenylated GDP-Rabs (7). A family of small integral membrane proteins related to Yip3/PRA, despite promiscuous binding to prenylated Rabs, shows partially specific GDF activity *in vitro*(8,9).

A second group of candidate-targeting determinants are RabGEFs which promote formation of Rab-GTP and thus prevent Rab-GDP membrane extraction mediated by RabGDI. To date, only a small number of RabGEFs have been identified, including Sec2 for Sec4 (10), Varp for Rab21 (11), Rabin3 for Rab8 (12), Rabex5 for Rab5 and Rab21 (13,14), Rab3GEP for Rab3 and Rab27 (15,16) and the connecdenns for Rab35 (17). They are structurally diverse proteins that do not contain a common catalytic core akin to the TBC (Tre-2, Bub2 and CDC16) domain of RabGAPs although groups of GEFs do contain Vps9 (13) or DENN domains (18). RabGEFs have been proposed as targeting factors for their substrate Rabs. Sec2 affects the distribution of Sec4, and Varp plays a role in Rab21 localization to early endosomes (11), but their precise function in Rab targeting remains unclear. Finally, effector engagement could play a role in targeting by stabilizing Rab membrane association. An example is the Rab9 effector tail interacting protein of 47 kD (TIP47), which contributes to the steady-state localization of Rab9 to late endosomes, and is able to partially redirect Rab1/9 chimeras to late endosomes (19).

In this study, we use Rab27a as a model Rab to test mechanisms of Rab membrane recruitment. Rab27a controls the motility of melanosomes in melanocytes (20–23) and the exocytosis of secretory granules in regulated secretory cells such as cytotoxic T-lymphocytes (CTLs), mast cells and pancreatic β cells (24,25). The recruitment of diverse effector molecules by Rab27a accounts for the performance of these distinct functions in different cell types. To date, 11 Rab27 effectors have been identified which can be classified into 4 subgroups. Most contain a conserved N-terminal Rab27 binding domain (R27BD). These include Mlph/Slac2a and Myrip/Slac2c which act as adaptors linking Rab27 to myosin molecular motors, synaptotagmin-like proteins (Slp) 1–5 which contain tandem C2 domains at the C-terminus, and effectors shared with Rab3 such as Rabphilin3A (26). Munc13-4, implicated in lytic granule docking in CTLs (27), is the only Rab27 effector identified that lacks the N-terminal R27BD.

In melanocytes, Rab27a localizes to melanosomes and its role in melanosome transport is well characterized. Only a subset of Rab27 effectors has been reported to be expressed in melanocytes, namely Mlph and Slp2 (28–30). Activated Rab27a forms a tripartite complex with Mlph and MyoVa which allows the anchoring of melanosomes to the actin cytoskeleton at the cell periphery. In the absence of any of the three components of the complex, loss of peripheral tethering leads to the clustering of melanosomes in the perinuclear area, as observed in melanocytes from the mouse mutants *ashen* (Rab27a-deficient), *leaden* (Mlph-deficient) and *dilute* (MyoVa-deficient) (31). Recently, we identified Rab3GEP, previously isolated as a Rab3a GEF (16), as the non-redundant Rab27GEF in melanocytes (15). Rab3GEP depletion in these cells leads to clustering of melanosomes in the perinuclear region of the cell, with the loss of Rab27a activation resulting in a similar phenotype to that of loss of Rab27a expression in *ashen* melanocytes.

The extensive characterization of Rab27a and its accessory proteins in this system makes melanosome transport an ideal model for investigating the mechanisms governing Rab localization to a specific subcellular membrane compartment.

## Results

### Generation of a Rab27a chimeric mutant unable to interact with effectors

To test the role of Rab27a effector engagement in targeting Rab27a to melanosomes we systematically introduced mutations into Rab27a in order to disrupt its ability to interact with effector molecules. We replaced the Rab family (RabF) and Rab subfamily (RabSF) regions, proposed sites of effector binding (32), with the corresponding sequences from other Rab proteins (Figure S1). This strategy was adopted to minimize the risk of compromising the global structure of the mutant Rab27a molecules. The ability of the resulting chimeras to interact with Rab27a effector molecules was tested using the yeast two-hybrid system. Our panel of interactor proteins was representative of all four subgroups of effectors: the Rab27a-specific Slac2 and Slp proteins, and the effectors shared with Rab3a, which all contain a conserved N-terminal Rab27-binding domain (R27BD), and Munc13-4 which binds Rab27a through a distinct domain located in the medial region of the protein (Table 1).

**Table 1:** Interaction of Rab27a mutants with known Rab27a effectors

	Mlph	MyRIP	Rabphilin3A	Slp1	Slp2	Slp3	Slp4	Slp5	Munc13-4
Rab27a WT	++	++	++	++	++	++	++	++	++
Rab3 Q81L	−	−	+	−	−	−	+	−	−
Rab5 WT	−	−	−	−	−	−	−	−	−
Rab27a^F4^ (FFRDAM to YYRGAM)	−	−	−	++	++	−	++	++	−
Rab27a FF88,89YY	−	++	++	++	++	++	++	++	−
Rab27a D91G	+	++	−	++	++	+	++	++	−
Rab27a^SF1^ (YDYLIKF to FDYMFKI)	−	++	++	++	++	++	++	++	++
Rab27a Y6F	−	++	++	++	++	++	++	++	++
Rab27a L9M	++	++	++	++	++	++	++	++	++
Rab27a I10F	++	++	++	++	++	++	++	++	++
Rab27a F12I	++	++	++	++	++	++	++	++	++
Rab27a^SF2^ (NSKFIT to HEFQES)	++	++	++	++	++	++	++	++	++
Rab27a^SF1/F4^	−	−	−	−	−	−	−	−	−

Interaction of Rab27a mutants with effectors was tested using a yeast two-hybrid β-galactosidase assay. ++ denotes positive result in β-galactosidase assay after 4 h, + denotes positive result after 24 h, − denotes negative result after 24 h.

**Figure 1:**
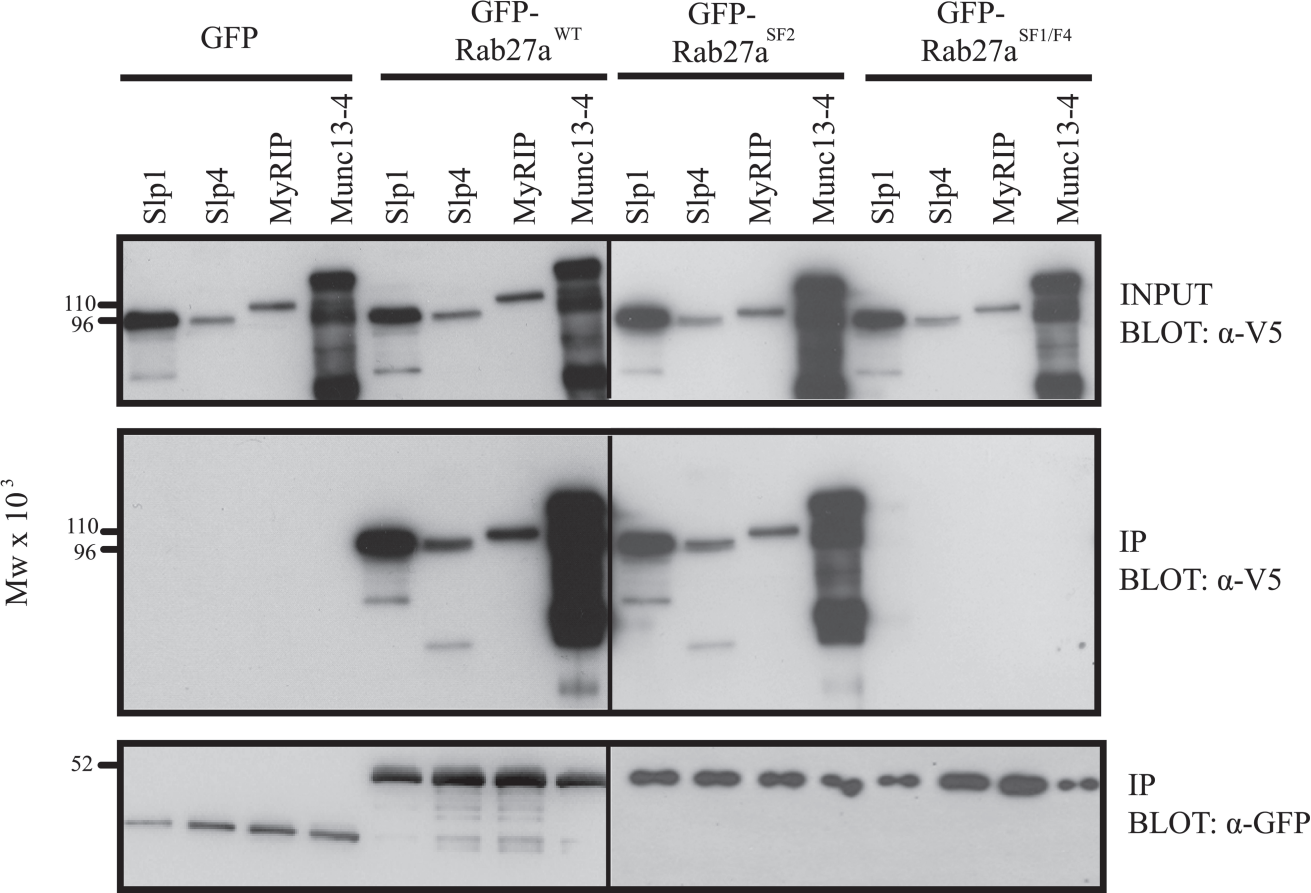
Rab27a^SF1/F4^ is unable to bind effectors, whereas Rab27a^SF2^ retains effector-binding capacity HEK293A cells co-expressing the indicated GFP-Rab27a mutants and V5 effectors were lysed and subjected to immunoprecipitation with α-GFP antibodies. Immune complexes were harvested using sheep α-rabbit dynabeads and immunoprecipitates were probed for the presence of V5-tagged effectors with mouse α-V5 antibodies and GFP-Rab27a mutants with mouse α-GFP antibodies.

Briefly, we found that the Rab27a^F4^ chimera (Rab27a FFRDAM to Rab3a YYRGAM) ablated interaction with the R27BD of Mlph, MyRIP, Rabphilin3A, Slp3 and full-length Munc13-4, but retained interaction with the Slp1, 2, 4 and 5 R27BDs. Point mutations within the Rab27a F4 region suggested that the YY sequence was important for Mlph binding while the D residue was important for Rabphilin3A interaction (Table 1). The Rab27a^SF1^ chimera (Rab27a YDYLIKF to Rab3a FDYMFKI) resulted only in lost interaction with Mlph and point mutations revealed that Rab27a Y6 is the key residue in this region. Strikingly, the combination mutant Rab27a^SF1/F4^, where both the Rab27a F4 and SF1 regions were replaced, was unable to interact with any of the effectors tested, despite expression levels comparable to those of wild-type (WT) Rab27a (Figure S2). Conversely, the Rab27a^SF2^ chimera (Rab27a NSKFIT to Rab5a HEFQES) retained binding to representatives of all four Rab27a effector subgroups. Binding specificity in the assay was confirmed using Rab5a, which does not interact with any Rab27 effector, Rab27a WT which interacts with all effectors and Rab3a Q81L which interacts only with Rabphilin3A and Slp4 as controls. The yeast two-hybrid results were confirmed by heterologous expression of GFP-Rab27a mutants and V5-tagged effectors representing the four Rab27a effector subgroups in HEK293A cells followed by Rab27a immunoprecipitation using α-GFP antibody. WT Rab27a and Rab27a^SF2^ were able to interact with V5-tagged Slp1, Slp4, MyRIP and Munc13-4. However, Rab27a^SF1/F4^ was unable to interact with any of the effectors tested, consistent with the results from the yeast two-hybrid assay. GFP alone was unable to interact with any effectors, confirming that binding was Rab27a-specific (Figure 1).

To verify the usefulness of the Rab27a^SF1/F4^ chimera as a tool, we analysed its prenylation status as a sensitive measure of Rab protein folding. Extraction with Triton-X-114 showed that EGFP-Rab27a^SF1/F4^ expressed in HEK293A cells partitioned into the detergent phase to a similar level as EGFP-Rab27a WT, indicating lipid modification and thus correct folding (Figure S3). Rab27a^SF2^ was also efficiently prenylated, while Rab27a^SGS^, where the prenylatable cysteine residues were replaced with serines, remained in the aqueous phase as expected.

**Figure 2:**
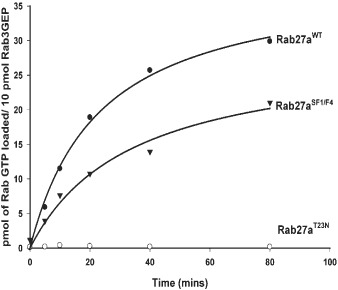
Rab27a^SF1/F4^ can bind GTP and is a substrate for Rab3GEP GST-Rab27a WT (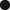), GST-Rab27a^SF1F4^ (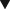) and GST-Rab27T23N (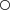) were incubated with ^35^S-GTPγS at 30°C for the indicated periods of time in the presence of recombinant Rab3GEP. Stimulation of ^35^S-GTPγS binding was quantified by filter-binding assay followed by scintillation counting. Each reaction contained 80 pmol of Rab. The data shown are the mean of duplicate determinations from a single experiment, which is representative of three such experiments. Results were plotted after subtraction of control values obtained in the absence of Rab3GEP.

Lack of effector binding by Rab27a^SF1/F4^ could be due to a failure to bind GTP. However, an *in vitro* GTP exchange assay using recombinant Rab3GEP and his_6_-tagged Rab27a proteins demonstrated that Rab27a^SF1/F4^ was a substrate for Rab3GEP and could be loaded with GTP (Figure 2). Rab27a T23N, a GDP-restricted mutant, was unable to bind GTP as expected. The results suggest that deficiencies in GTP binding are not responsible for the inability of Rab27a^SF1/F4^ to interact with effectors.

### Subcellular localization and function of Rab27a chimeras in melanocytes

**Figure 3:**
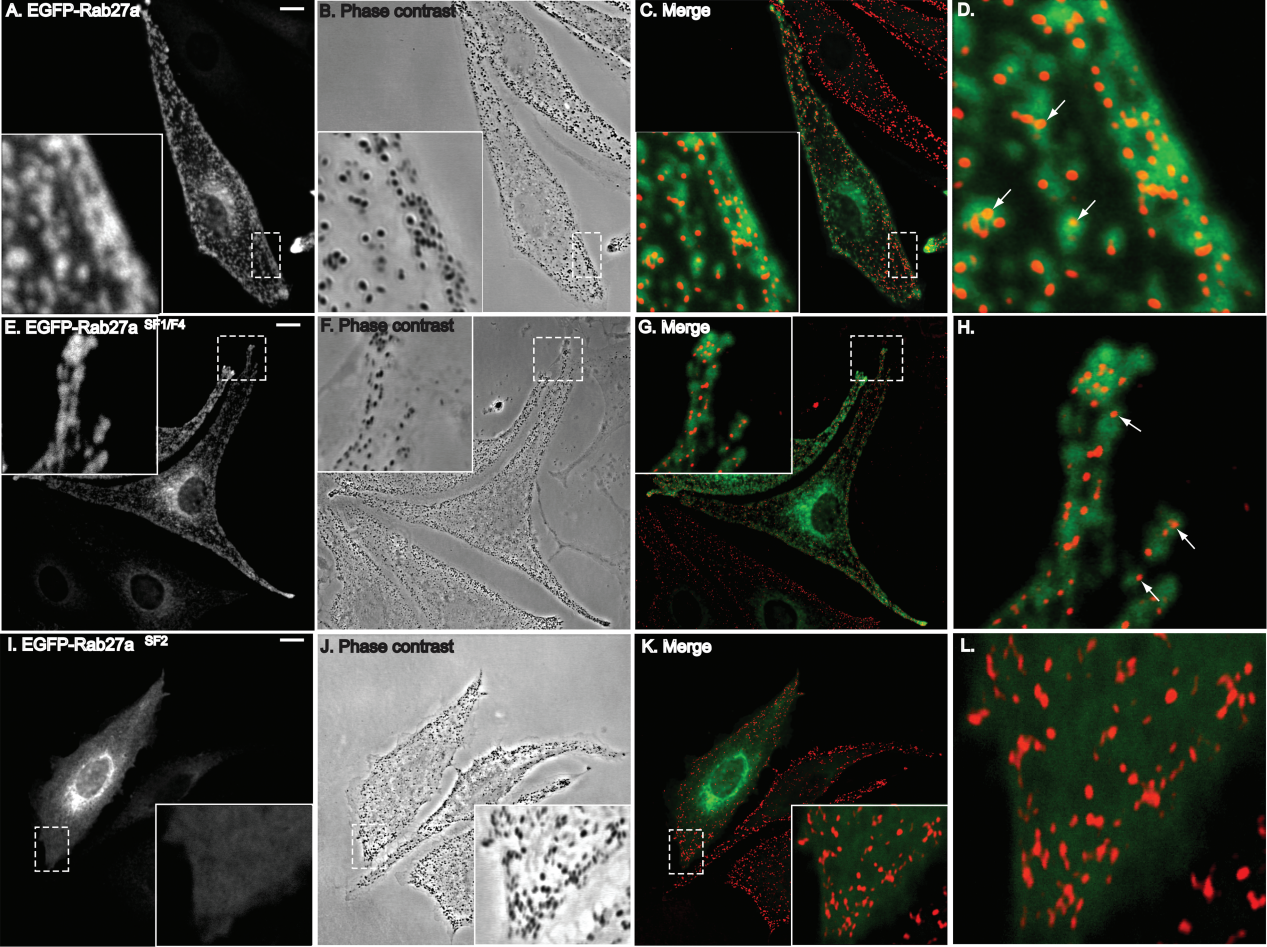
Expression of chimeric EGFP-Rab27a proteins in WT melanocytes WT melanocytes were transiently transfected with EGFP-Rab27a WT (A–D), EGFP-Rab27a^SF1F4^ (E–H) or EGFP-Rab27a^SF2^ (I–L). Cells were fixed and observed by confocal microscopy (A, E and I) and phase contrast (B, F and J). In panels C, G and K the merged images show the overlap between EGFP fluorescence (green) and pigmented melanosomes (red pseudocolour). Insets are magnifications of the indicated regions showing subcellular localization of Rab27a mutants in more detail. Panels D, H and L are blow-ups of the insets in panels C, G and K. Bar = 10 µm.

To examine the link between effector binding and melanosome targeting, EGFP-tagged Rab27a chimeras were expressed in melanocytes. EGFP-Rab27a WT was observed in a characteristic punctate pattern and colocalized extensively with melanosomes at the cell periphery as expected (Figure 3). Additionally, the Rab27a^SF1/F4^ chimera retained the ability to localize to melanosomal membranes while the Rab27a^SF2^ chimera failed to target to melanosomes and mislocalized to endoplasmic reticulum (ER)/Golgi membranes as described previously (33). To analyse the localization of Rab27a^SF1/F4^ in more detail, it was compared directly to WT Rab27a. EGFP-tagged Rab27a^SF1/F4^ and mRFP-tagged Rab27a WT colocalized extensively on melanosomal membranes, similar to what was observed with EGFP- and mRFP-tagged WT Rab27a. Rab27a^SF2^ did not colocalize with Rab27a WT on melanosomes (Figure S4). It is noteworthy that the expression of Rab27a chimeras in WT melanocytes did not alter melanosome distribution, demonstrating that these mutations do not confer a dominant negative effect.

**Figure 4:**
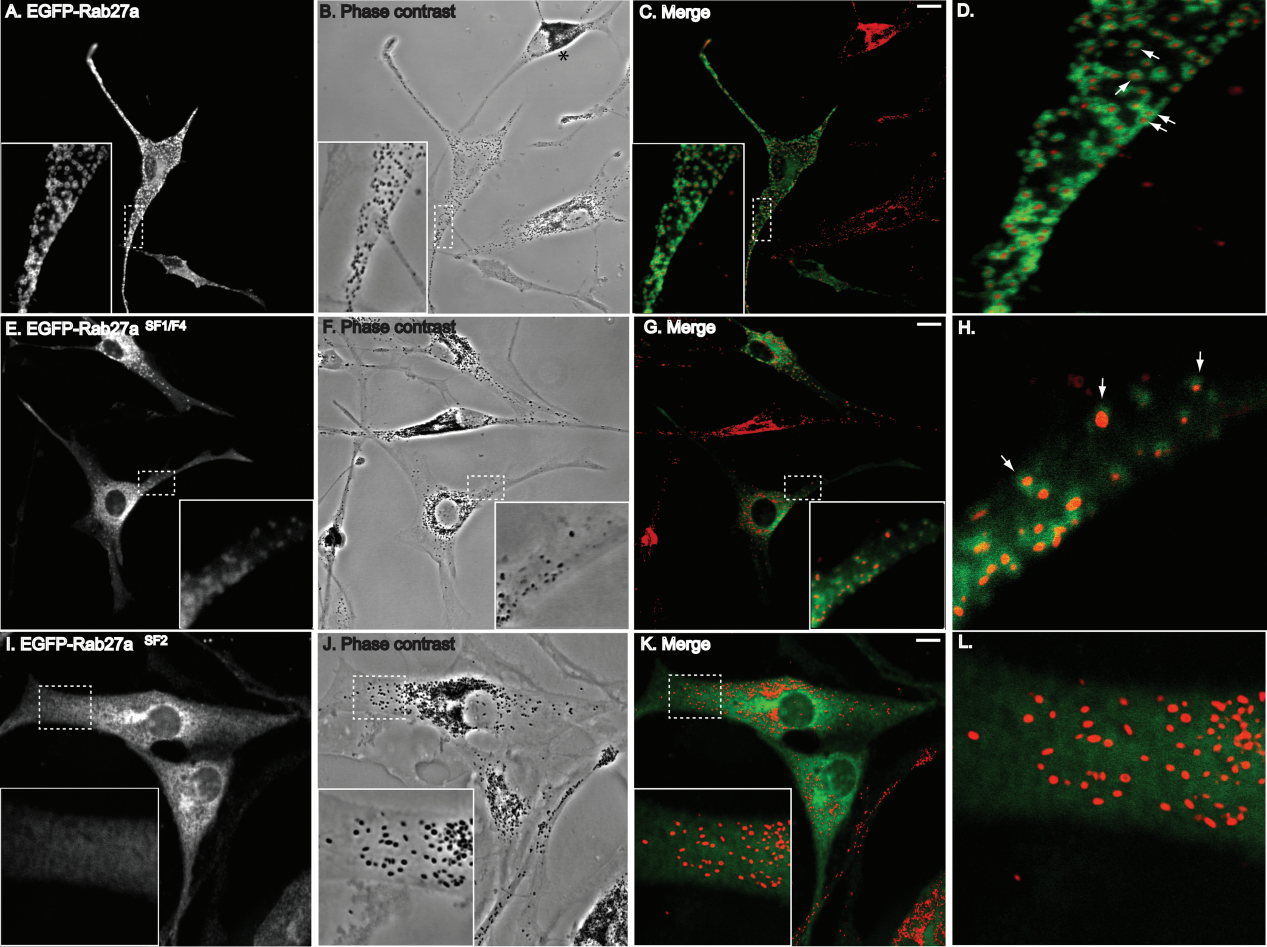
Expression of chimeric EGFP-Rab27a proteins in melan-*ash2* (Rab27a null) melanocytes Melan-*ash2* melanocytes were transiently transfected with EGFP-Rab27a WT (A–D), EGFP-Rab27a^SF1F4^ (E–H) or EGFP-Rab27a^SF2^ (I–L). Cells were fixed and observed by confocal microscopy (A, E and I) and phase contrast (B, F and J). In panels C, G and K the merged images show the overlap between EGFP fluorescence (green) and pigmented melanosomes (red pseudocolour). Insets are magnifications of the indicated regions showing subcellular localization of Rab27a mutants in more detail. Panels D, H and L are blow-ups of the insets in panels C, G and K. * indicates untransfected cell. Bar = 10 µm.

In *ashen* (Rab27a null) mutant melanocytes, the lack of endogenous Rab27a leads to clustering of melanosomes in the perinuclear region of the cell. Transient expression of EGFP-Rab27a WT in these cells rescues the normal peripheral distribution of melanosomes, confirming that the introduced protein is both correctly targeted and functionally active (Figure 4). EGFP-Rab27a^SF1/F4^ correctly localized to melanosomes in *ashen* cells but, consistent with its inability to interact with Mlph in the yeast two-hybrid assay, did not rescue the clustering phenotype. This suggests that Rab27a^SF1/F4^ contains all the requisite targeting information irrespective of its inability to engage effectors. Rab27a^SF2^ mislocalized to ER/Golgi membranes in *ashen* melanocytes, as seen in WT cells. No rescue of the *ashen* phenotype was observed despite the ability of Rab27a^SF2^ to interact with effectors. These observations indicate that the lack of functional activity is due to loss of targeting information, which cannot be compensated for by effector binding.

A possible reason for Rab27a^SF1/F4^ inability to function in *ashen* melanocytes could be due to effects on membrane stability. To test this, the dynamics of EGFP-Rab27a^SF1/F4^ was examined in *ashen* melanocytes using fluorescence recovery after photobleaching (FRAP). We observed that melanosome-associated EGFP-Rab27a^SF1/F4^ fluorescence, like EGFP-Rab27a^WT^, did not recover within 5 min of photobleaching (data not shown). This indicates that loss of effector interaction does not affect the stability of Rab27a within the membrane.

### The Rab27a GEF Rab3GEP is necessary for Rab27a localization to melanosomes

**Figure 5:**
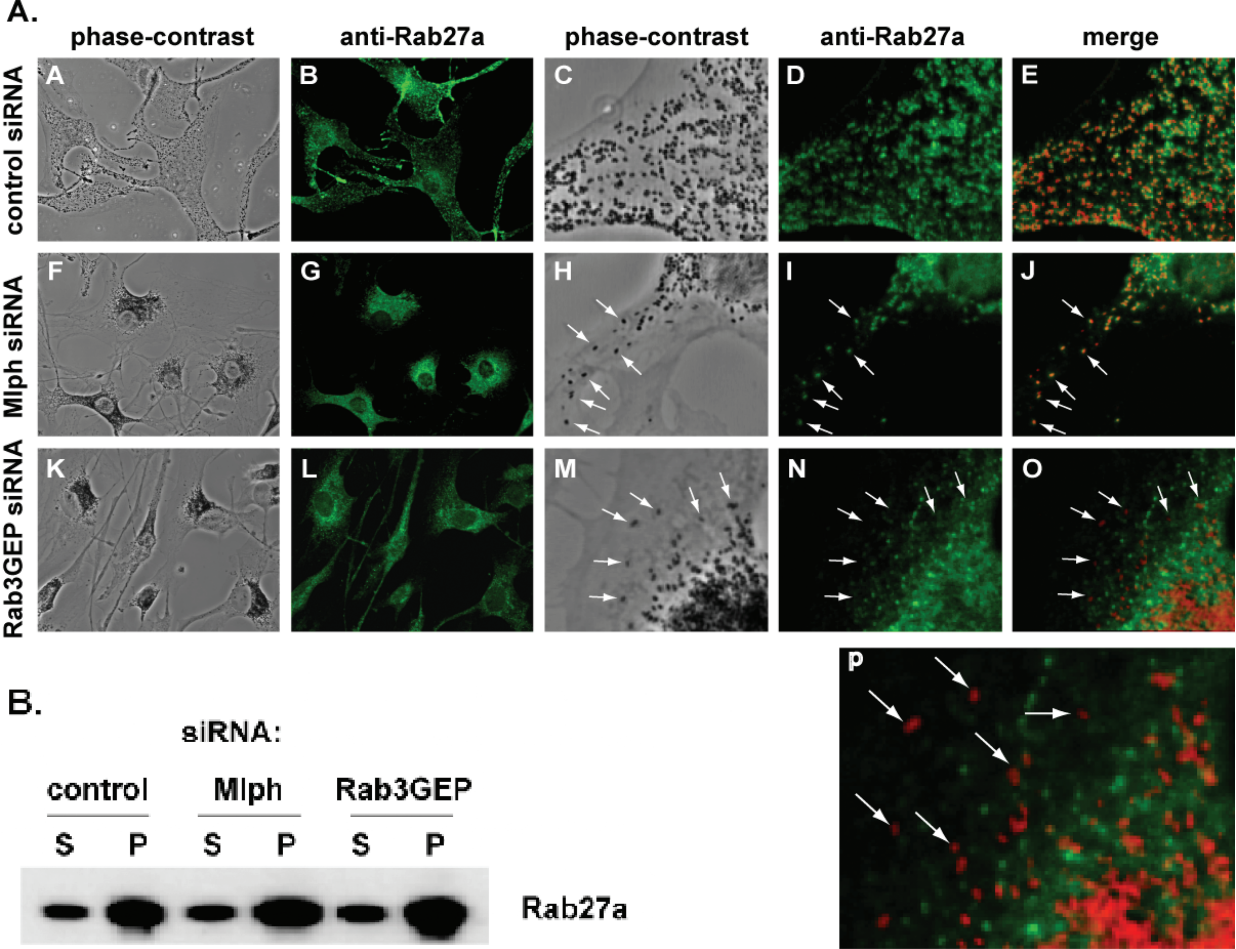
Endogenous Rab27a mislocalizes in Rab3GEP-depleted melanocytes A) WT melanocytes transfected with either non-targeting siRNA oligos (A–E), Mlph-specific siRNA oligos (F–J) or Rab3GEP-specific siRNA oligos (K–O) were fixed 72 h after transfection and labelled with antibodies to Rab27a. Panels A, F and K show phase contrast images depicting the distribution of melanosomes. Panels B, G and L depict the distribution of Rab27a. Panels C–E, F–J and K–O show overlap between Rab27a and melanosomes under these conditions at higher magnification. Panel P is a blow-up of panel O. Arrows mark the positions of individual pigmented melanosomes. B) Melanocytes were transfected with siRNAs as above. After 72 h, cells were disrupted and separated into soluble (S) and pelletable (P) fractions by centrifugation at 100 000 ×***g***. Rab27a was detected by immunoblotting.

We next tested the role of GEF activity in Rab27a targeting. Rab3GEP is the non-redundant exchange factor for Rab27a in melanocytes. Depletion of Rab3GEP with siRNAs leads to the perinuclear aggregation of melanosomes because of an inability to activate Rab27a, as described previously (15). We labelled Rab3GEP-depleted melanocytes with antibodies to Rab27a to investigate its subcellular localization. Efficiently depleted cells were identifiable on the basis of melanosome clustering. As shown in Figure 5A, Rab27a was no longer detected on mature melanosomes, indicating that Rab3GEP plays an important role in its targeting. As a control, we induced melanosome clustering by depleting melanocytes of Mlph. Rab27a was readily observed on melanosomes that had lost their peripheral distribution because of Mlph depletion. In Rab3GEP-depleted melanocytes, Rab27a was found on membraneous structures distinct from melanosomes. Subcellular fractionation confirmed that Rab27a was predominantly membrane-bound in these cells, similar to what is seen in cells treated with non-targeting or Mlph-specific siRNAs (Figure 5B).

**Figure 6:**
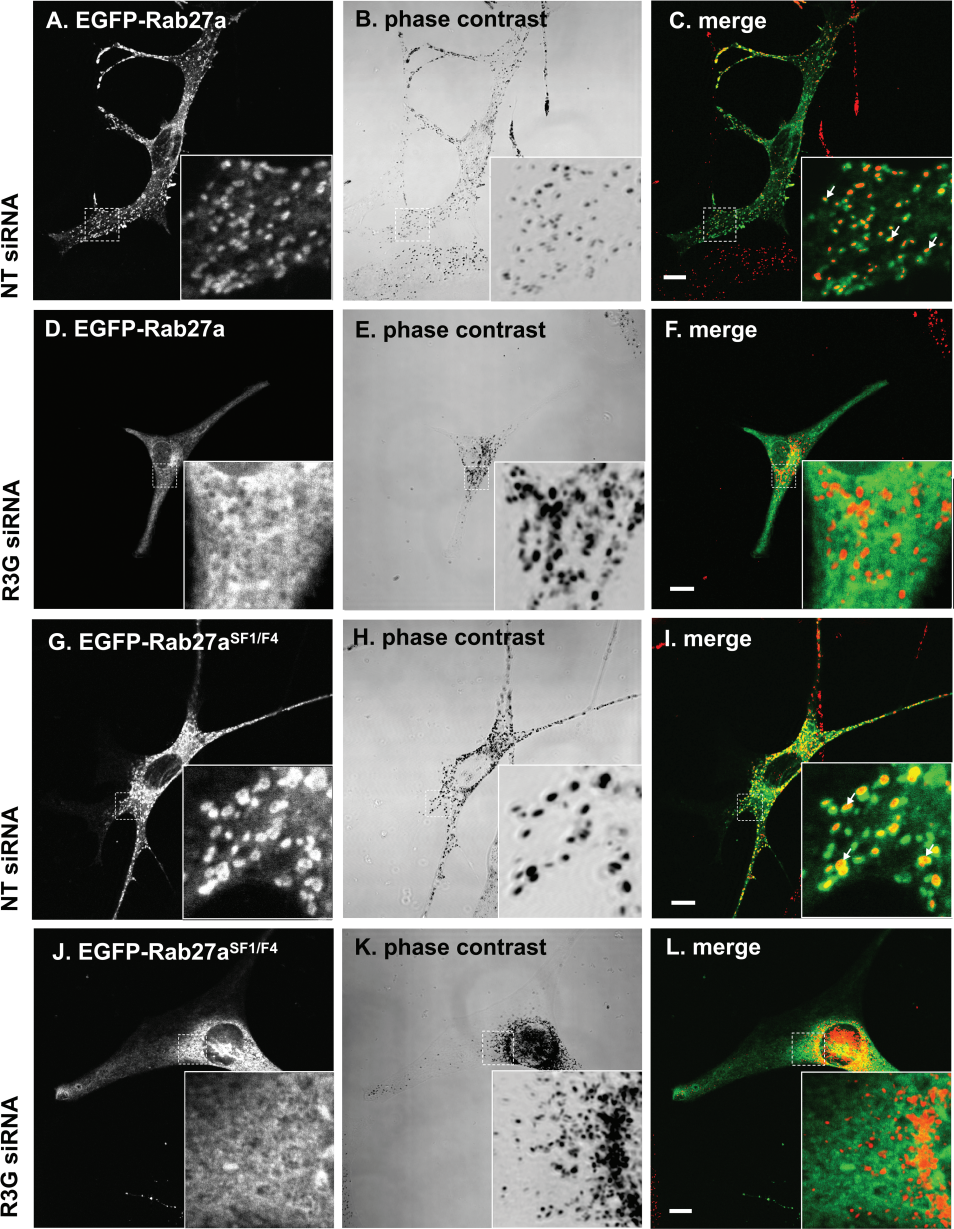
Localization of EGFP-Rab27a WT and EGFP-Rab27a^SF1/F4^ mutants in Rab3GEP-depleted melanocytes WT melanocytes treated with control non-targeting or Rab3GEP-specific siRNA oligos for 72 h were transiently transfected with EGFP-Rab27a WT or EGFP-Rab27a^SF1F4^, as indicated. Cells were fixed and observed by confocal microscopy and phase contrast. Insets show selected regions at higher magnification.

To extend these findings, we analysed the melanosomal targeting of newly synthesized Rab27a in Rab3GEP-depleted cells. Melanocytes were treated with control or Rab3GEP siRNAs, and subsequently transfected with EGFP-Rab27a WT or EGFP-Rab27a^SF1/F4^. In cells treated with control siRNA, both Rab27a WT and Rab27a^SF1/F4^ clearly localized to melanosomes (Figure 6). However, in cells lacking Rab3GEP, both Rab27a WT and Rab27a^SF1/F4^ were mislocalized to non-melanosomal membranes. Taken together, these data suggest that Rab3GEP, while not required for the association of Rab27a with membranes *per se*, is necessary for its correct localization to melanosomes.

### Rab3GEP is not sufficient for Rab27a localization to melanosomes

**Figure 7:**
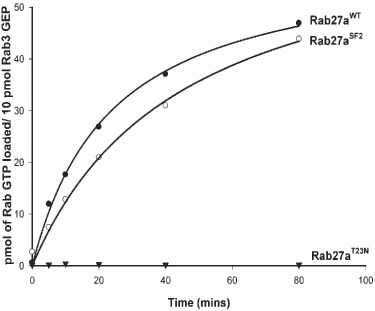
Rab27a^SF2^ can bind GTP and is a substrate for Rab3GEP GST-Rab27a WT (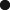), GST-Rab27a^SF2^ (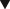) and GST-Rab27a^T23N^ (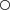) were incubated with ^35^S-GTPγS at 30°C for the indicated periods of time in the presence of recombinant Rab3GEP. Stimulation of ^35^S-GTPγS binding was quantified by filter-binding assay followed by scintillation counting. The data shown represent the mean of duplicate determinations from a single experiment, which is representative of three such experiments. Each reaction contained 80 pmol of Rab. Each curve is the result of subtraction of control values obtained in the absence of Rab3GEP.

Rab27a^SF2^ is unable to localize correctly to melanosomes despite its ability to undergo prenylation and interact with known Rab27a effectors. Given the role of Rab3GEP in the targeting of Rab27a, a potential explanation for the failure of Rab27a^SF2^ to localize correctly may be that it is not a substrate for Rab3GEP-mediated nucleotide exchange. We tested this hypothesis using an *in vitro* exchange assay with recombinant his_6_-Rab27a^SF2^ and his_6_-Rab3GEP. Rab27a^SF2^ bound GTP to a similar extent as Rab27a^WT^ in the presence of Rab3GEP, and is thus able to be activated efficiently by Rab3GEP (Figure 7). Rab27a T23N was unable to bind GTP as expected. This finding is consistent with the ability of Rab27a^SF2^ to interact with effector molecules as GTP binding is a pre-requisite for effector recruitment. Another mis-targeted Rab27a chimera, Rab27a^SF3^ (Rab27a VRNWIS to Rab5a AKNWVK) was described previously (33) and is similarly membrane-associated and a substrate for Rab3GEF (unpublished observation). The characteristics of these mis-targeted mutants suggest that Rab3GEP, although necessary, is not sufficient for targeting of Rab27a to melanosomes, and raises the possibility that other, as yet unidentified, factors are required in conjunction with Rab3GEP for correct localization.

## Discussion

In this study, we examined the targeting of Rab27a to melanosomes as a model to dissect the mechanisms responsible for the specificity of Rab GTPase recruitment to target membranes. We describe an essential role for the Rab27a nucleotide exchange factor Rab3GEP, but also show that its activity is not sufficient. Additionally, we find no evidence for the involvement of known Rab27a effectors, which suggests a requirement for additional specificity-determining targeting factors.

To characterize the interaction of Rab27a with its effectors, we constructed a series of Rab27a/Rab3a chimeras, where the Rab27a RabF and RabSF regions were replaced with the corresponding regions from Rab3a. Their ability to interact with effector proteins was assessed by yeast two-hybrid assay. The Rab27a^F4^ mutant was unable to interact with Mlph, MyRIP, Rabphilin3A, Slp3 and Munc13-4. Rab27a^SF1^ had only lost binding to Mlph. However, the Rab27a^SF1/F4^ combination mutant was unable to interact with any effector tested. In contrast, Rab27a^SF2^ was able to interact with all the effectors tested. These findings are consistent with recent X-ray crystallographic analyses of Rab27 in complex with Mlph and Slp2a R27BD (34,35). Both these studies implicated the Rab27a SF1 and F4 regions in the interaction with Mlph and Slp2. In particular, Y6, F88 and D91 were identified as contact points for Mlph. This was verified by the point mutations generated in our study, which extended these findings by suggesting an important role for the Rab27a SF1 and F4 regions in the interaction with all Rab27a effectors containing the N-terminal R27BD. Furthermore, we show that these regions are also critical for binding to Munc13-4. This protein represents a different class of Rab27 effector that interacts with Rab27a via a poorly defined 300-amino acid region which bears no direct sequence homology to the N-terminal R27BD of the Slp/Slac2 family (30). Our data suggest that Rab27 binding to Munc13-4 is based on a similar mechanism to that defined for the interaction of Rab27 with Mlph.

Surface plasmon resonance analysis of the kinetics of Rab27 interaction with effectors suggests two distinct subsets: low-affinity effectors comprising Mlph, MyRIP and Slp3 and high-affinity effectors comprising Slp1, 2, 4 and 5 (36). We find that disruption of Rab27a F4 alone affects the interaction with the first group, whereas mutation of SF1 and F4 simultaneously is required to abrogate interaction with the second group as well. This suggests that binding to low-affinity effectors is lost when a single contact point in the F4 region is disrupted, while interaction with high-affinity effectors is only abolished by disruption of two contact points. Interestingly, Munc13-4 was unable to bind Rab27a^F4^. We therefore postulate that Munc13-4 may be a low-affinity effector, although further kinetic analysis is required to confirm this.

To assess the role of effector engagement in targeting of Rab27a, we examined the subcellular localization of Rab27a^SF1/F4^ in melanocytes. This effector binding-deficient mutant retained melanosomal localization. Conversely, Rab27a^SF2^, which retains the ability to interact with the known effectors tested, was mislocalized. Previous analysis of two Rab27a mutations, A152P and I44T, found in Griscelli syndrome type II patients revealed that loss of Mlph binding did not affect Rab27a targeting to melanosomes (37,38). However, Rab27a A152P retains the ability to interact with Slp1, 2, 4 and 5 (data not shown), and the I44T mutant retains interaction with Slp4 (37). Our study, using Rab27a^SF1/F4^, an effector binding-deficient mutant, extends these observations and suggests that effector binding is not a pre-requisite for Rab27a targeting to melanosomes. This is in contrast to the finding that the localization of Rab9 is at least in part determined by the Rab9 effector TIP47, which led to the idea that each Rab may be targeted by a ‘key effector’(19). Loss of TIP47 also led to destabilization of Rab9 (39). In contrast, Mlph is mislocalized and its expression levels are decreased in the absence of Rab27a, demonstrating that Rab27a recruits and stabilizes its effector Mlph (40). A better candidate for an effector with a role in targeting Rab27a may be Munc13-4, which localizes to lysosome-related organelles (LROs) independent of Rab27 (41). However, our study shows that neither Munc13-4 nor any of the other known Rab27a effectors are ‘key effectors'. Furthermore, our binding studies using two structurally unrelated classes of effectors raise the possibility that all Rab27a effectors may bind Rab27a through a similar mechanism. Hence, although we cannot formally rule out the possibility that another uncharacterized Rab27a effector could be involved in Rab27a localization, the present data argue against the generality of the ‘key effector’ hypothesis.

We next examined the role of nucleotide exchange in Rab27a targeting. Recently, we identified Rab3GEP, previously isolated as a GEF for Rab3, as the non-redundant exchange factor for Rab27a in melanocytes. In melanocytes lacking Rab3GEP, both endogenously and exogenously expressed Rab27a were mislocalized. Interestingly, Rab27a was not cytosolic in these cells, as might be expected for a GDP-bound Rab protein but was mis-targeted to perinuclear membranes and non-pigmented vesicles. The ability of Rab27a-GDP, in contrast to other GDP-bound which predominantly form cytosolic complexes with RabGDI, to associate stably with membranes allowed us to dissociate targeting and activation events clearly. Our data suggest that Rab3GEP is not necessary for membrane binding *per se* but is essential for correct localization to melanosomes. However, Rab3GEP nucleotide exchange activity is not sufficient, as shown by our Rab27a^SF2^ and Rab27a^SF3^ chimeras, which are mislocalized despite being substrates for Rab3GEP. This suggests that Rab3GEP mediates Rab27a localization in conjunction with other as yet unidentified targeting factors.

Mutations in the *Caenorhabditis elegans* homologue of Rab3GEP, AEX-3, have been shown to cause mislocalization of Rab3 from the synapse-rich axons to the cell body (42), suggesting that Rab3GEP could be involved in the targeting of its other Rab substrates. Another study has implicated Rab3GEP in the transport of synaptic vesicle precursors in Rab3a-positive vesicles by acting as a linker between Rab3a-GTP and Kif1 isoforms. Down-regulation of Rab3GEP in this system affects Rab3-positive vesicle localization although this is likely due to aberrant transport of vesicles rather than any effect on the initial targeting of Rab3a to the correct compartment (43).

Previous studies analysing the distribution of Rabs in the absence of GEF activity did not reach unequivocal conclusions. In temperature-sensitive mutants of the Sec4 GEF Sec2p, Sec4p was redistributed, suggesting that Sec2p may be required for targeting of Sec4p (10). However, Sec4p is still localized to post-Golgi vesicles, with vesicles themselves randomly distributed instead of localized to bud tips in the absence of functional Sec2p (10). Furthermore, the phenotype observed was similar to that seen in Myo2p (a MyosinVa ortholog) and actin mutants, suggesting that the secretory vesicles were mislocalized as a result of defects in actin tethering because of loss of Sec4p function, as is observed in melanocytes lacking Rab27a, rather than disruption of Sec4p membrane targeting. In contrast, the Rab21-specific GEF Varp appears to be required for endosomal localization of mammalian Rab21 in HeLa cells (11), and the Rab5 GEF Rabex-5 recruits an exogenously expressed GDP-bound mutant, Rab5a S35N, to endosomes, but its potential role in targeting WT Rab5 has not been tested (44). Our study adds to the growing evidence that RabGEFs could be important components in the machinery that regulates Rab targeting. Recently a systematic characterization of the 17 human DENN domain proteins revealed that they are specific GEFs for 10 Rabs (18). Our study raises the intriguing possibility that these DENN (differentially expressed in neoplastic versus normal cells)-containing GEFs could be involved in the targeting of their substrate Rabs as well as promoting nucleotide exchange.

Our data indicate that Rab3GEP activity is necessary but not sufficient for targeting Rab27a to melanosomes, suggesting that additional factors are required. This is supported by the finding that most RabGEFs identified to date are soluble proteins (10–12,14–17) and therefore presumably themselves require targeting to the correct membrane in order to activate Rabs. Future work should be directed at identifying factors potentially involved in the targeting of both Rabs and RabGEFs.

## Materials and Methods

### Plasmid constructs

Rab27a mutants described in this study were generated by site-directed mutagenesis (Quickchange, Strategene) using WT Rab27a cloned into yeast (pBTM), bacterial (pET14b) and mammalian (pEGFP) expression vectors as previously described (21,45,46). Construction of pGAD-Slp1 R27BD, Slp2a R27BD, Slp4a/granuphilin R27BD and Mlph R27BD and pGAD MyRIP R27BD has been described previously (46). pGAD Munc13-4 was PCR amplified from pENTR-Munc13-4 (kind gift of H. Horiuchi, Kyoto University) and cloned into a modified pGAD-C3 vector using *Cla*I and *Sal*I restriction sites. pGAD-Rabphilin3A R27BD was PCR amplified from pGEX-Rabphilin3A (kind gift of T. Sudhof) and subcloned into pGAD using *Eco*RI/*Sal*I restriction sites. pGAD Slp3 and Slp5 R27BD were PCR amplified from pEF-T7 Slp3 and pEF-T7 Slp5, respectively (kind gift of M. Fukuda, RIKEN), and subcloned into pGAD using *Eco*RI/*Sal*I and *Pst*I/*Bgl*II restriction sites, respectively. Preparation of pFastBacHT-B-Rab3GEP and pET14b-Rab27a vector was described previously (15). pRFP-Rab27a was generated by amplifying Rab27a from pEGFP-Rab27a and subcloning into pRFP-C. Primer sequences are available on request. pENTR-V5-MyRIP has been described previously (47). pENTR-V5-Slp1 was generated by PCR amplification of Slp1 from pEF-T7 Slp1 with primers containing *Eco*RI/*Xho*I restriction sites and the product cloned into pENTR-V5 restricted with *Eco*RI/*Sal*I. pENTR-V5-Slp4 was generated by PCR amplification of Slp4 from IMAGE clone BC014913 and the product cloned into pENTR-V5 using *Eco*RI/*Sal*I restriction sites. pENTR-V5-Munc13-4 was generated by PCR amplification of Munc13-4 from pENTR-Munc13-4 and the product cloned into pENTR-V5.

### Antibodies

Anti-Rab27a monoclonal antibody 4B12, described previously (21), was used at 1:10 000 dilution for immunoblot. Anti-Rab27a polyclonal antibody (21) was used at 1:100 dilution for immunofluorescence. Other antibodies were used at the following dilutions for immunoblot: mouse monoclonal anti-GFP (Roche) 1:1000, mouse monoclonal anti-LexA (Santa Cruz) 1:1000.

### Cell culture

Mouse WT melanocytes (melan-ink4a) and melan-ink4a-*ashen* (melan-*ash2*) (21) were maintained in RPMI 1640 supplemented with 10% foetal calf serum, 2 mm L-Glutamine, 100 U/mL penicillin, 100 U/mL streptomycin, 200 nm Phorbol-1,2-myristate-1,3-acetate and 200 pm Cholera Toxin. HEK293A cells were maintained in DMEM supplemented with 10% foetal calf serum, 2 mm L-Glutamine, 100 U/mL penicillin and 100 U/mL streptomycin. All cells were cultured at 37°C under a humidified atmosphere containing 10% CO_2_.

### Yeast two-hybrid assays

All yeast media and strains were as described previously (46). Yeast L40 strain was transformed by lithium acetate procedure with combinations of pBTM and pGAD constructs and grown for 2 days on standard drop-out medium plates lacking tryptophan and leucine. Colonies were streaked out in patches, grown for 2 days and assayed for β-galactosidase activity as described previously (46).

### Sf9 virus production

The production of Rab3GEP recombinant baculovirus was performed according to the manufacturer's instructions (Invitrogen) and as described previously (15).

### Recombinant proteins

Recombinant his_6_-tagged, Rab27a and Rab27a mutants were expressed in *Escherichia coli* BL21-codon plus (DE3) RILP (Stratagene) and purified on a nickel–Sepharose column by fast protein liquid chromatography (FPLC) as described previously (4). Recombinant his_6_-Rab3GEP was prepared from Sf9 cell pellets using nickel–Sepharose affinity chromatography as described for his tagged Rabs.

### Immunoblot

Protein samples were separated on 10% or 12.5% SDS–PAGE gels and transferred to polyvinylidene fluoride (PVDF) membrane (Millipore). Membranes were blocked in 5% non-fat dried milk, 0.2% (v/v) Tween-20 in PBS (PBS-T) for 1 h, incubated with primary antibody diluted in 1% non-fat dried milk in PBS-T, washed three times with PBS-T, incubated with horse radish peroxidase labelled secondary antibody (anti-mouse or anti-rabbit, Dako, 1:10 000) and then washed as above. Bound antibody was detected using the ECL Plus Western Blotting detection system (Amersham).

### Triton-X-114 partition

Triton-X-114 partition was performed as described previously (45). Briefly, lysates obtained from cells transfected with the appropriate vector were adjusted to 1% Triton-X-114 and placed on ice for 5 min. Samples were then incubated at 37°C for 5 min, and the phases were separated by centrifugation at 20 000 ×***g*** for 5 min. The detergent phase (lower) was removed and the aqueous phase was readjusted to 1% Triton-X-114 and the above-mentioned procedure was repeated. The two detergent phase fractions were combined and the volume adjusted to equal that of the aqueous phase. SDS–sample buffer was added to both phases and samples were boiled for 5 min. Equivalent amounts of detergent and aqueous phases were loaded onto 10% SDS–PAGE gels, electrophoresed, blotted onto PVDF membranes, and detected with anti-GFP monoclonal antibodies.

### Immunoprecipitation

For immunoprecipitation, HEK293A cells were co-transfected with GFP-Rab27a mutants and V5-tagged effectors. Cells were allowed to express exogenous proteins for 48 h and then lysed into buffer A (20 mm Tris pH 7.5, 150 mm NaCl, 1 mm DTT, 5 mm GTP, 1% CHAPS and 1× protease inhibitor cocktail). Following centrifugation for 10 min at 800 ×***g***, cell lysates were incubated with 2 µg of rabbit α-GFP for 4 h at 4°C before addition of 50 µL of sheep anti-rabbit dynabeads (Dynal, Invitrogen) and incubation for 2 h at room temperature with end-over-end rotation. Immune complexes were then harvested using the Dynal magnetic particle collector, washed three times using buffer A, subjected to SDS–PAGE on a 12.5% gel, and immunoblotted using mouse antibodies specific for V5 and GFP.

### Transfection

Melanocytes or HEK293A cells were seeded onto glass coverslips at the appropriate density, and transfected the next day. Cells were transfected with plasmid DNA using FuGENE6 (Roche) and serum-free medium OptiMEM I (Invitrogen) according to the manufacturer's protocol. Per 16-mm well 0.5 µg of plasmid DNA and 1.5 µL of FuGENE6 were used. siRNA oligonucleotide transfection was performed using Oligofectamine (Invitrogen) and cells were incubated for 72 h post-transfection. Oligofectamine was used at 0.625 µL per 16-mm well or 2.5 µL per 35-mm well. The final concentration of siRNA was 100 nm. All siRNA oligos were purchased from Dharmacon. For Rab3GEP, sequences were as follows: duplex1-gcgagaagacgacaccauuuu; duplex2-gcccagacccacuacuauauu. A pool of four non-targeting control siRNAs was used as a negative control, and a melanophilin-specific siRNA (15) as a positive control.

### Immunofluorescence

Transfected cells were processed for immunofluorescence 24–72 h after transfection. Cells on coverslips were rinsed with PBS, fixed with 3% (w/v) paraformaldehyde at room temperature for 15 min, rinsed three times with PBS, and permeabilized with buffer A (0.5% BSA and 0.05% saponin) for 15 min. Coverslips were incubated with the primary antibody diluted in buffer A for 90 min, washed with buffer A, incubated with Alexa 488- and/or Alexa 568-conjugated secondary antibodies (Molecular Probes) followed by buffer A washes. The coverslips mounted with ImmunoFlour mounting medium were subjected to fluorescence and phase contrast microscopy using a DM-IRBE confocal microscope (Leica) fitted with 40× 1.0 NA (numerical aperture) oil-immersion Fluotar objective lens. All images are single sections in the *z*-plane. Images were processed with leica tcs-nt and Adobe Photoshop software.

### GDP/GTP exchange assay

Rab3GEP activity was assayed by measuring the association of ^35^S-GTPγS (Amersham) with Rab27a WT and mutants. For time–course exchange assays, 480 pmol of His-Rabs were incubated at 30°C with 0 or 60 pmol of recombinant Rab3GEP in a 300 µL reaction mixture containing 50 mm Tris–HCl pH 7.5, 12 mm MgCl_2_, 2 mm ethylenediaminetetraacetic acid (EDTA), 0.2 mg/mL BSA, 0.6 pmol ^35^S-GTPγS (Amersham) and 959.4 pmol GTPγS (Fluka) (1562.5 d.p.m./pmol). At each time-point, a 50-µL aliquot was removed and the reaction was stopped by snap-freezing in liquid nitrogen. Protein samples were applied to nitrocellulose filters (Whatman) pre-equilibrated in ice-cold wash buffer (50 mm Tris–HCl pH 7.5, 10 mm MgCl_2_, 0.2 mg/mL BSA). Filters were washed twice with 2 mL ice-cold wash buffer. Protein-bound radioactivity was determined by scintillation counting.

## Supporting Information

**Figure S1: Schematic representation of Rab27a mutants.** Schematic representation of Rab27a mutants generated in this study. Residues in red are mutated. F = RabFamily regions and SF = rabsubfamily regions.

**Figure S2: Expression levels of LexA-Rab27a/Rab3a chimeras in L40 yeast.** L40 yeast transformed with the appropriate construct were lysed as described in *Materials and Methods* and the lysate separated on a 12.5% SDS–PAGE gel, transferred to PVDF and immunoblotted using α-LexA antibody.

**Figure S3: Rab27a^SF1*&sol;*F4^ and Rab27a^SF2^ are efficiently prenylated.** EGFP-Rab27a mutants were expressed in HEK293A cells for 24 h. The cells were lysed by repeated passage through a 26-gauge needle. The lysate was adjusted to 1% Triton-X-114 and the aqueous and detergent phases separated. Equivalent volumes of detergent and aqueous phase were loaded onto a 12.5% SDS–PAGE gel, transferred to PVDF and immunoblotted with α-GFP antibody. TCL = total cell lysate, DET = detergent phase, Aq = aqueous phase. Quantification of % Rabin detergent phase was measured by dividing band intensity in DET lane by total intensity in DET + Aq lanes.

**Figure S4: Subcellular localization of Rab27a^SF1F4^ and Rab27a^SF2^ mutants.** WT melanocytes were transiently co-transfected with mRFP-Rab27a and either EGFP-Rab27a (A–D), EGFP-Rab27a^SF1F4^ (E–H) or EGFP-Rab27a^SF2^ (I–L). Cells were fixed and observed by confocal microscopy. EGFP fluorescence (A, E, I), mRFP fluorescence (B, F, J) and merged fluorescent images (C, J, K) with colocalization represented in yellow are shown. Panels D, H and L depict the corresponding brightfield images; insets show higher magnifications.
