# Supplementary material for: Rab27a Targeting to Melanosomes Requires Nucleotide Exchange but Not Effector Binding
Source: Traffic. 2011 Jun 13;12(8):1056–66. doi: 10.1111/j.1600-0854.2011.01216.x (PMC3509405; doi:10.1111/j.1600-0854.2011.01216.x)
Supplement: Supplementary file 1 [file tra0012-1056-SD1.pdf]

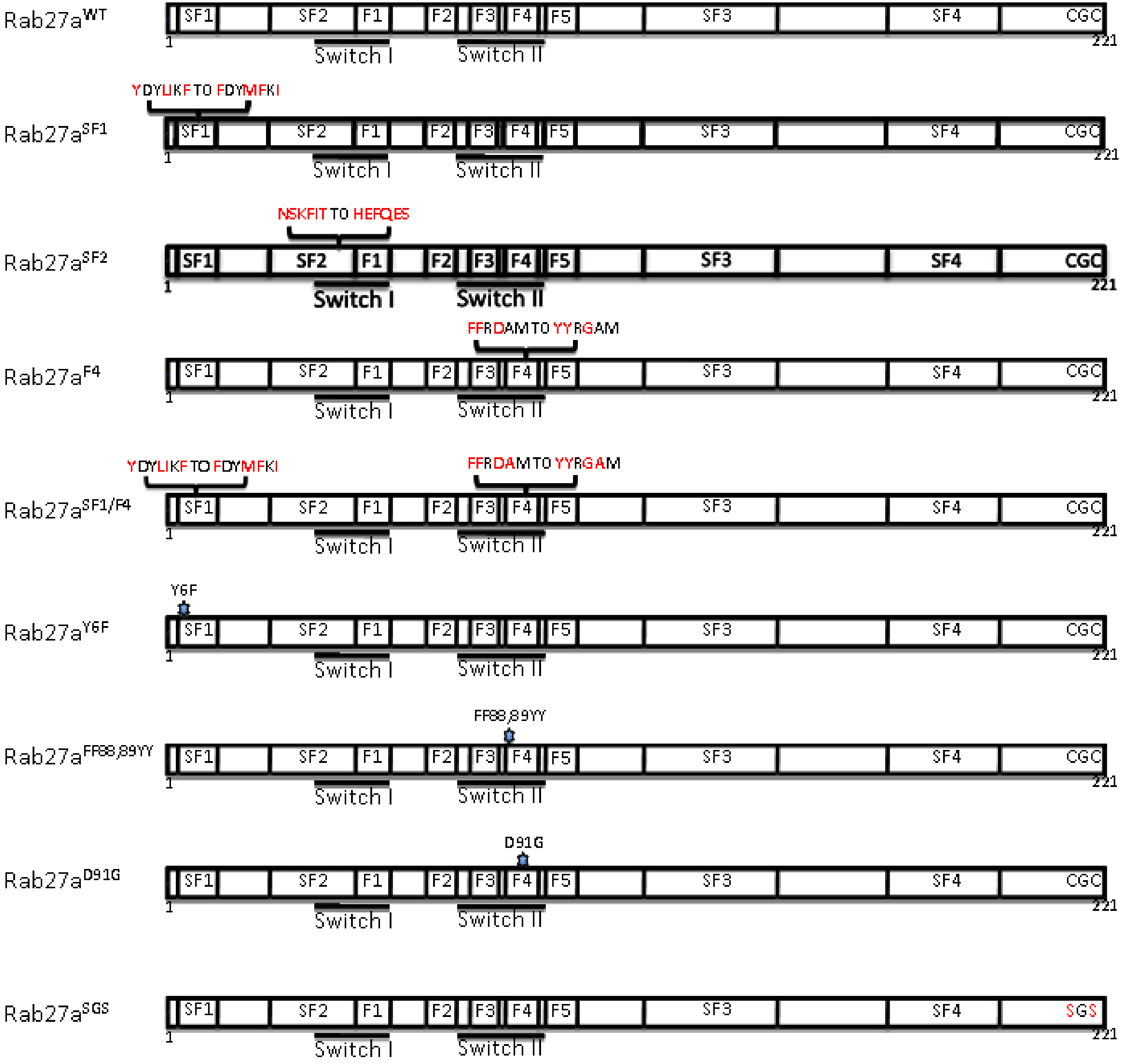

Supplementary figure 1. Schematic representation of Rab27a mutants. Schematic representation of Rab27a mutants generated in this study. Residues in red are mutated. F = RabFamily regions and SF= rabsubfamily regions
