# Supplementary material for: Rab27a Targeting to Melanosomes Requires Nucleotide Exchange but Not Effector Binding
Source: Traffic. 2011 Jun 13;12(8):1056–66. doi: 10.1111/j.1600-0854.2011.01216.x (PMC3509405; doi:10.1111/j.1600-0854.2011.01216.x)
Supplement: Supplementary file 2 [file tra0012-1056-SD2.pdf]

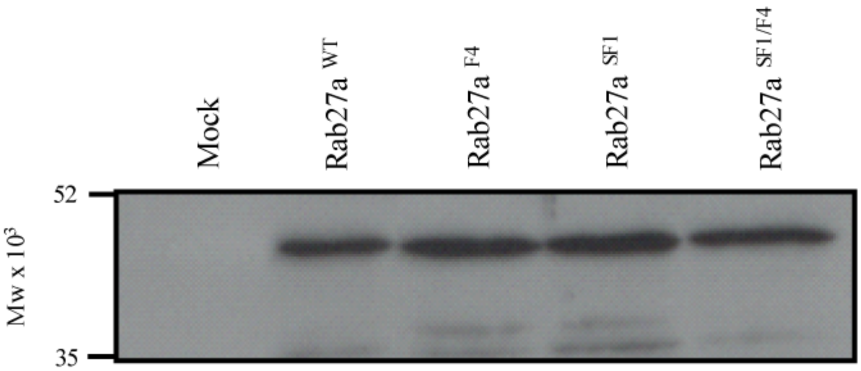

Supplementary Figure 2. Expression levels of LexA-Rab27a/Rab3a chimeras in L40 Yeast. L40 Yeast transformed with the appropriate construct were lysed as described in 'Materials and Methods' and the lysate separated on a 12.5 % SDS-PAGE gel, transferred to PVDF and immunoblotted using α-LexA antibody.
