# Supplementary material for: Rab27a Targeting to Melanosomes Requires Nucleotide Exchange but Not Effector Binding
Source: Traffic. 2011 Jun 13;12(8):1056–66. doi: 10.1111/j.1600-0854.2011.01216.x (PMC3509405; doi:10.1111/j.1600-0854.2011.01216.x)
Supplement: Supplementary file 4 [file tra0012-1056-SD4.pdf]

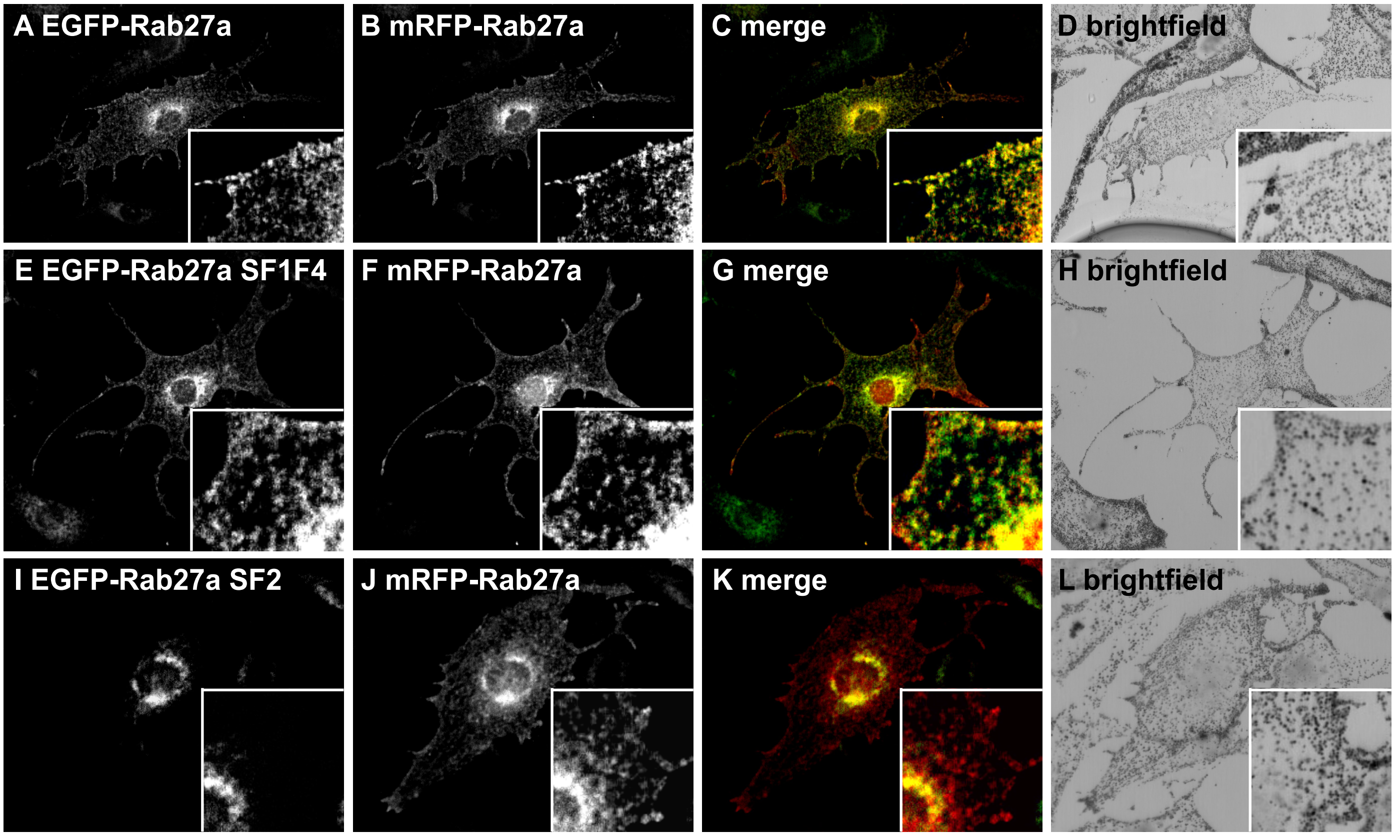

Supplementary Figure 4. Subcellular localisation of Rab27a^{SF1F4} and Rab27a^{SF2} mutants. Wild-type melanocytes were transiently co-transfected with mRFP-Rab27a and either EGFP-Rab27a (A-D), EGFP-Rab27a^{SF1F4} (E-H) or EGFP-Rab27a^{SF2} (I-L). Cells were fixed and observed by confocal microscopy. EGFP fluorescence (A, E, I), mRFP fluorescence (B, F, J) and merged fluorescent images (C, J, K) with co-localisation represented in yellow are shown. Panels D, H and L depict the corresponding brightfield images; insets show higher magnifications.
